# Supplementary material for: Hamster organotypic modeling of SARS-CoV-2 lung and brainstem infection
Source: Nat Commun. 2021 Oct 4;12:5809. doi: 10.1038/s41467-021-26096-z (PMC8490365; doi:10.1038/s41467-021-26096-z)
Supplement: Supplementary file 1 — Supplementary Information [file 41467_2021_26096_MOESM1_ESM.pdf]

Supplementary information

Hamster organotypic modeling of SARS-CoV-2 lung and brainstem infection

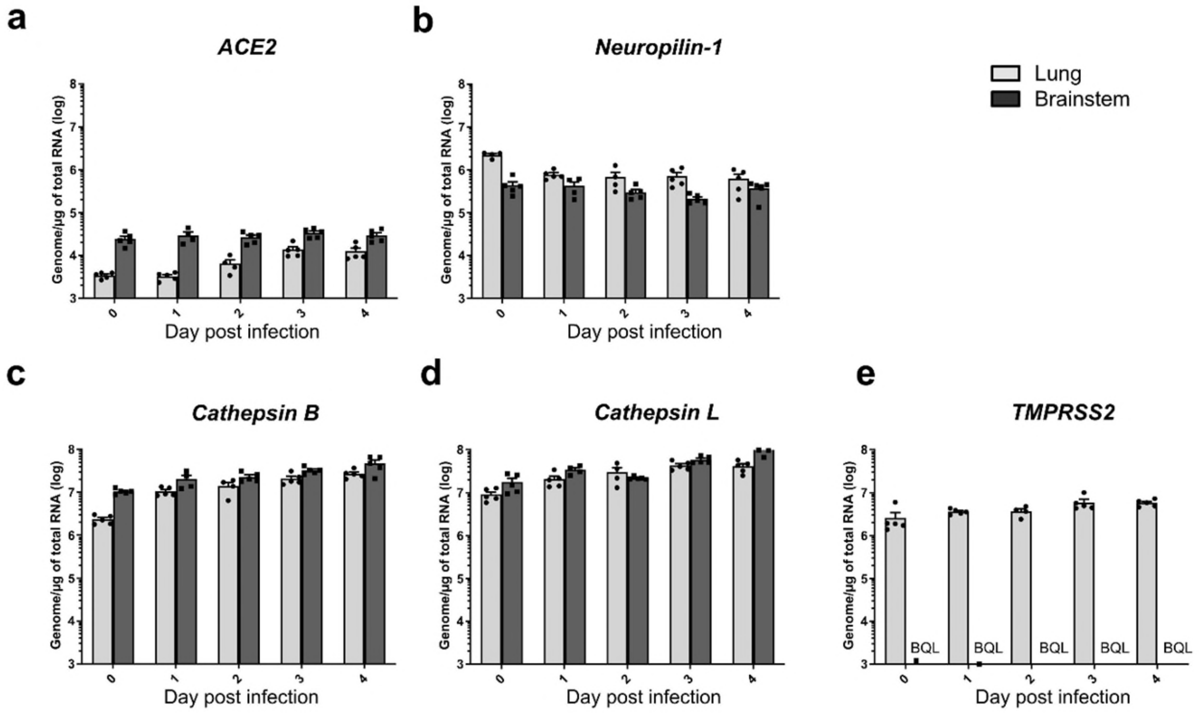

f

mRNA copies per 10,000 cells

	Neurons	Oligodendrocytes	Astrocytes	Microglia
ACE2	3,56E+02	3,74E+02	1,46E+02	3,50E+02
Neuropilin-1	8,29E+04	2,94E+04	5,09E+03	9,54E+04
Cathepsin B	1,43E+06	6,06E+05	1,63E+05	1,64E+06
Cathepsin L	1,19E+06	1,21E+06	2,21E+05	1,15E+06
TMPRSS2	BQL	BQL	BQL	BQL

Supplementary figure 1. Characterization of the mRNA expression of ACE2, Neuropilin and serine proteases in organotypic cultures.

a,b,c,d,e, ACE2, Neuropilin-1 and serine protease mRNA expression quantified by RT-qPCR in organotypic cultures infected with 1,000 PFU of SARS-CoV-2. n= minimum 4 biologically independent animals over 2 independent experiments. **f,** Neurons, oligodendrocytes, astrocytes and microglia from non-infected cerebellar cultures at day 0 were sorted by fluorescence-activated single cell sorting using respectively anti-gaba-A receptor, anti-GalC, anti-GLAST and anti-CD68 antibodies. ACE2, Neuropilin-1 and serine proteases mRNA copies/10^4 cells were quantified by RT-qPCR in each cell populations. BQL: below quantification limit; ACE2: Angiotensin-converting enzyme 2; PTFE: Polytetrafluoroethylene; GalC: Galactosylceramidase. Error bars represent SD. Source data are provided as a Source Data file.

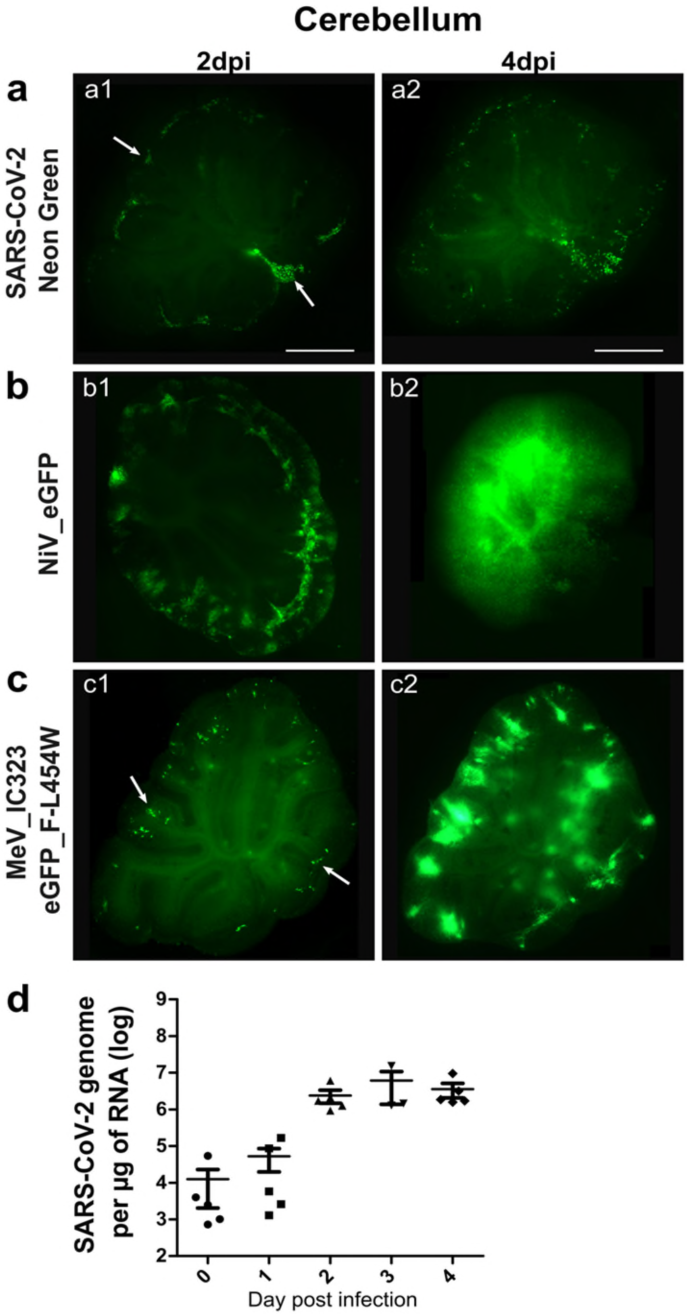

Supplementary figure 2. Dissemination of three respiratory viruses including SARS-CoV-2 in hamster organotypic cerebellar cultures.

Comparative entry and spread of 3 different respiratory viruses showing encephalitic properties: **a,** icSARS-CoV-2-mNG (infection: 10,000 pfu), **b,** NiV-EGFP (infection: 5,000 pfu) and **c,** the hyperfusogenic variant MeV IC323-EGFP-F L454W (infection: 1000 pfu) were monitored by following the fluorescence at 2 dpi and 4 dpi in cerebellum slices. Pictures were taken using a Nikon Eclipse Ts2R microscope, reconstituted using the Stitching plug-in with ImageJ software (Preibisch et al., 2009) and are representative of 3 independent experiments. Scale bar = 1 mm. **d,** SARS-CoV-2 genomes per µg of total RNA were quantified by RT-qPCR in cerebellum organotypic cultures (n=5 biologically independent animals) at 90min post infection and 1, 2, 3 and 4 days post infection (dpi) with 5,000 pfu and normalized to the standard deviation for GAPDH mRNA. Source data are provided as a Source Data file.

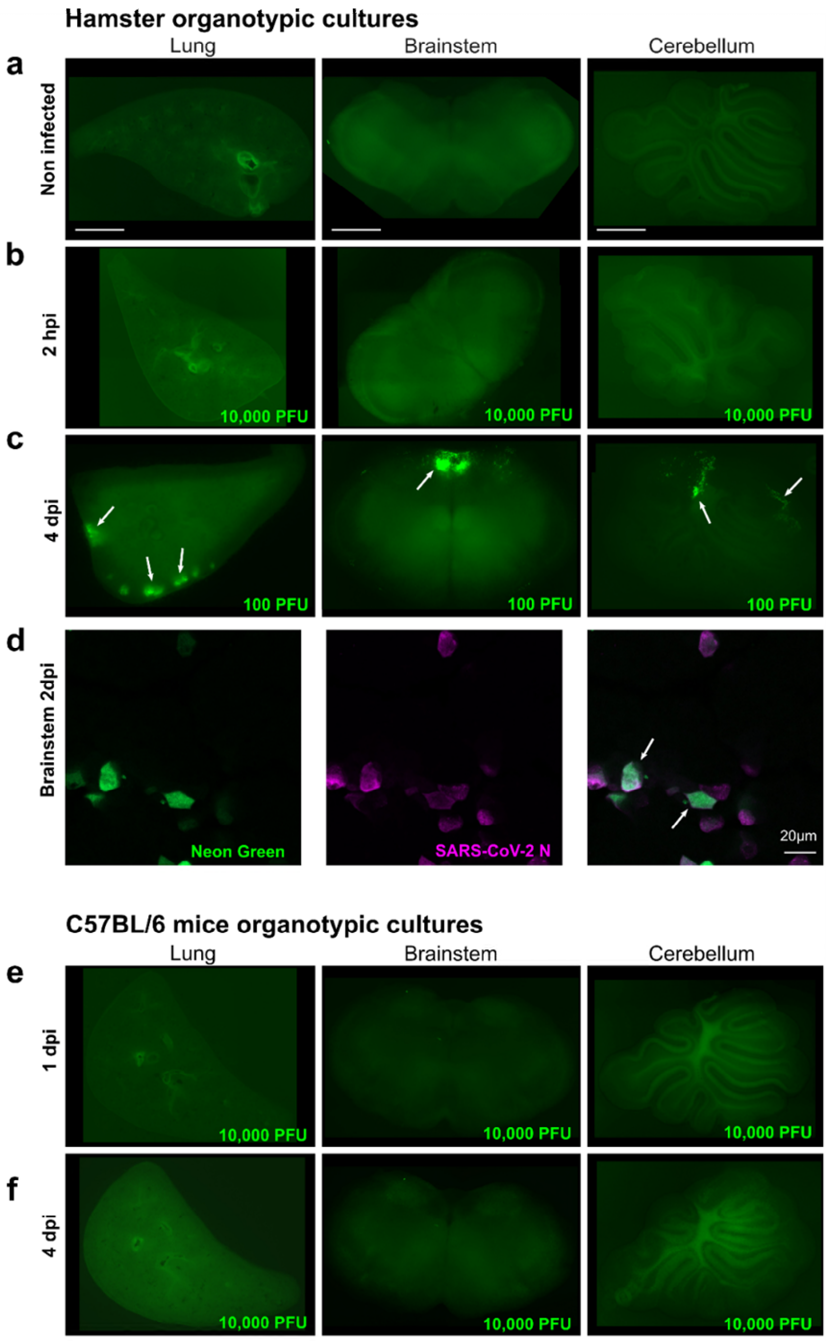

Supplementary figure 3. Controls for the viral dissemination and the basal autofluorescence in SARS-CoV-2 infected and non-infected hamster and mice organotypic cultures.

Pictures of hamster organotypic cultures from lungs, brainstems and cerebella were taken **a,** 2 hours post contact with a vehicule (non-infected), **b,** 2 hours post infection (hpi) with 10,000 PFU of icSARS-CoV-2-mNG, and **c,** 4 days post infection (dpi) with 100 PFU of icSARS-CoV-2-mNG. **d**, Brainstem organotypic cultures were infected with 1000 PFU with icSARS-CoV-2-mNG and collected at 2 dpi. Immunofuorescent staining shows colocalization of Neon-green expressing cells and SARS-CoV-2 nucleoprotein (N) positive cells. **e,f,** C57BL/6 mice organotypic cultures from lungs, brainstems and cerebella were infected with 10,000 PFU of icSARS-CoV-2-mNG and viral entry was monitored by following the fluorescence at 1 dpi and 4 dpi. Pictures from **a,b,c,e,f,** were taken using a Nikon Eclipse Ts2R microscope and reconstituted using the Stitching plug-in with ImageJ software (Preibisch et al., 2009). Scale bar = 1 mm. The immunofluorescence staining analysis showed in **d,** was performed using a LSM800 confocal microscope from Zeiss. All images are representative of 3 independent experiments.

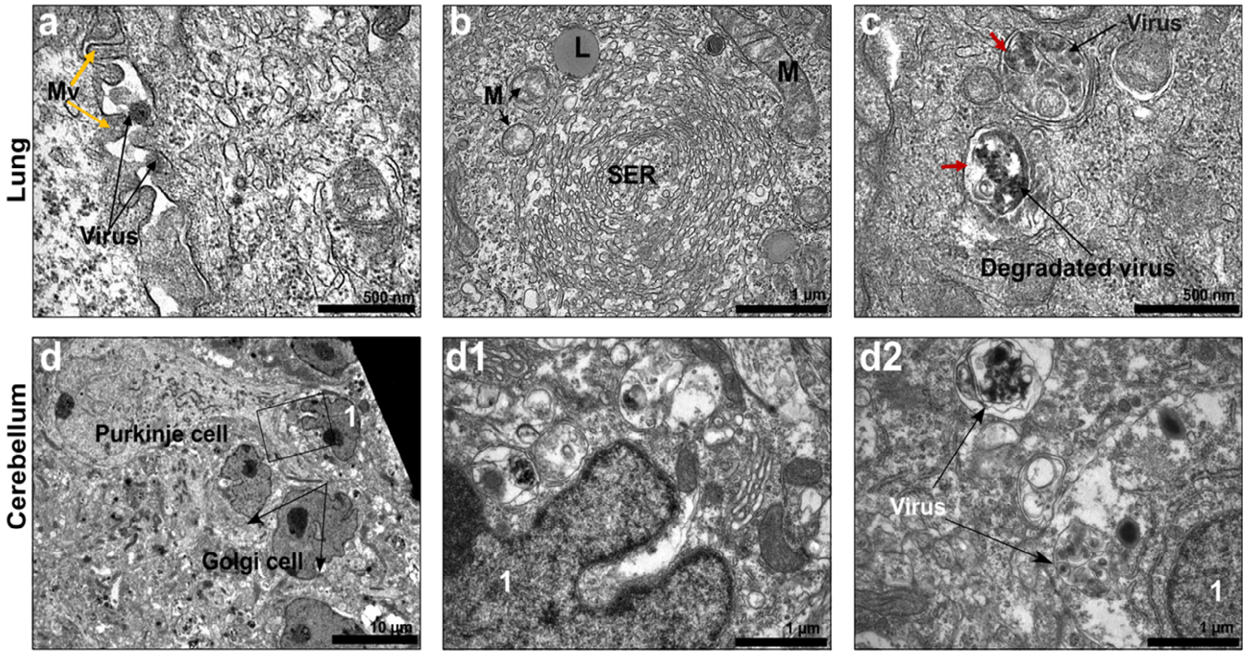

Supplementary figure 4. SARS-CoV-2 tropism in hamster organotypic lung and cerebellum cultures 1 and 2 days after infection.

Cultures were infected with 1,000 pfu of SARS-CoV-2 and fixed at 1 day post infection (organotypic lung cultures) or 2 days post infection (organotypic cerebellum cultures).

a,b,c, Ultrastructure of infected lung cells by transmission electron microscopy (TEM). **a,** High magnification of a type 2 pneumocytes apical surface with viral particles on the microvillus (Mv). **b,** Cytoplasm of a lung cell showing disorganization of the smooth endoplasmic reticulum (SER), the presence of lipid and mitochondria undergoing degradation. **c,** High magnification of the cytoplasm of a ciliated cell with several autophagosomes containing virions (arrows). Red arrows show the double membrane of the autophagosome. All images are representative of 2 independent experiments.

d, TEM analysis of cerebellum culture showing infected Golgi neurons close to an uninfected Purkinje neuron. **d1,2,** Enlargement of a Golgi neuron showing autophagosomes that contain viral particles (arrows).

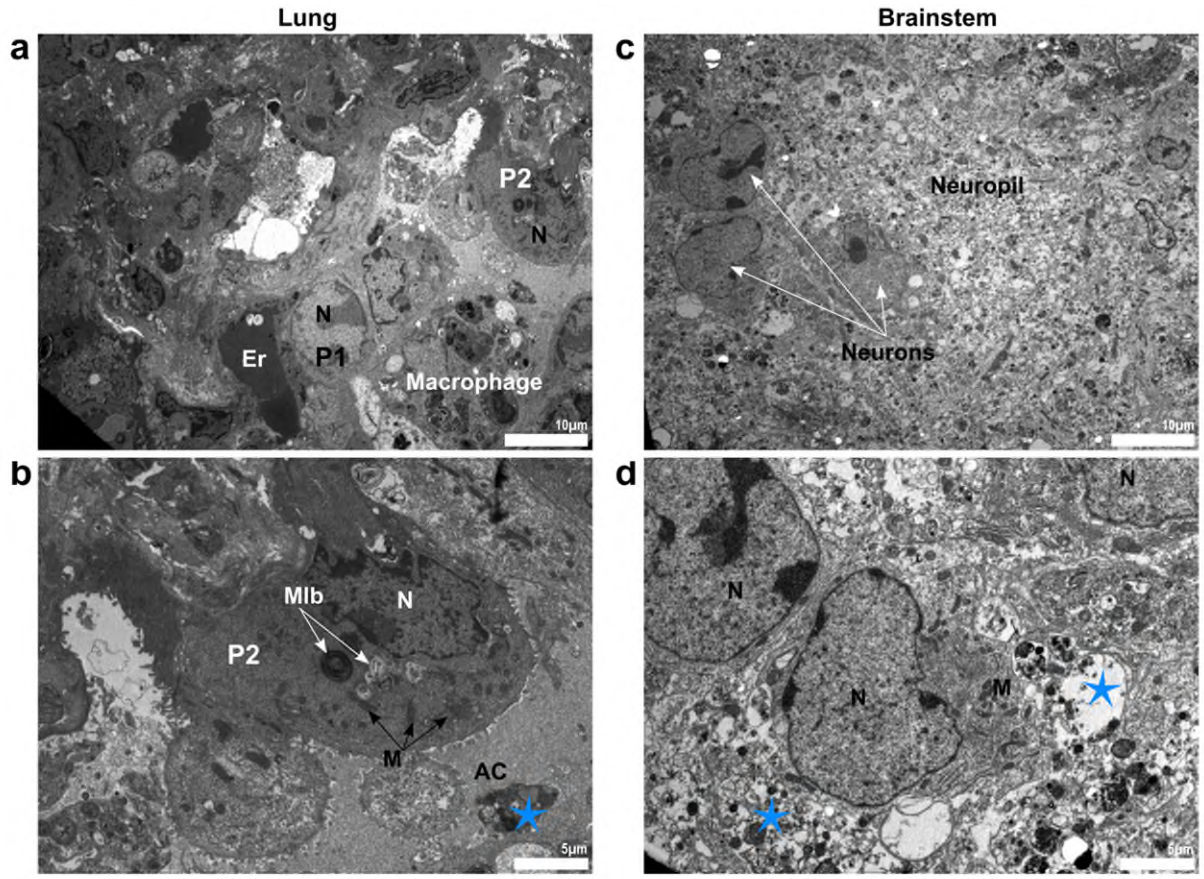

Supplementary figure 5. Non-infected hamster organotypic lung and brainstem cultures.

a,b, Ultrastructure of non-infected lung organotypic cultures by transmission electron microscopy (TEM) after 1 day of culture. **c,d,** Ultrastructure of non-infected brainstem organotypic cultures TEM after 2 days of culture. P1: type I pneumocyte; P2: type II pneumocyte with numerous mitochondria (M) and Multilamellar bodies (Mlb); AC: alveolar cavity; Er: erythrocyte; N: nucleus; Blue stars: cell debris. All images are representative of 3 independent experiments.

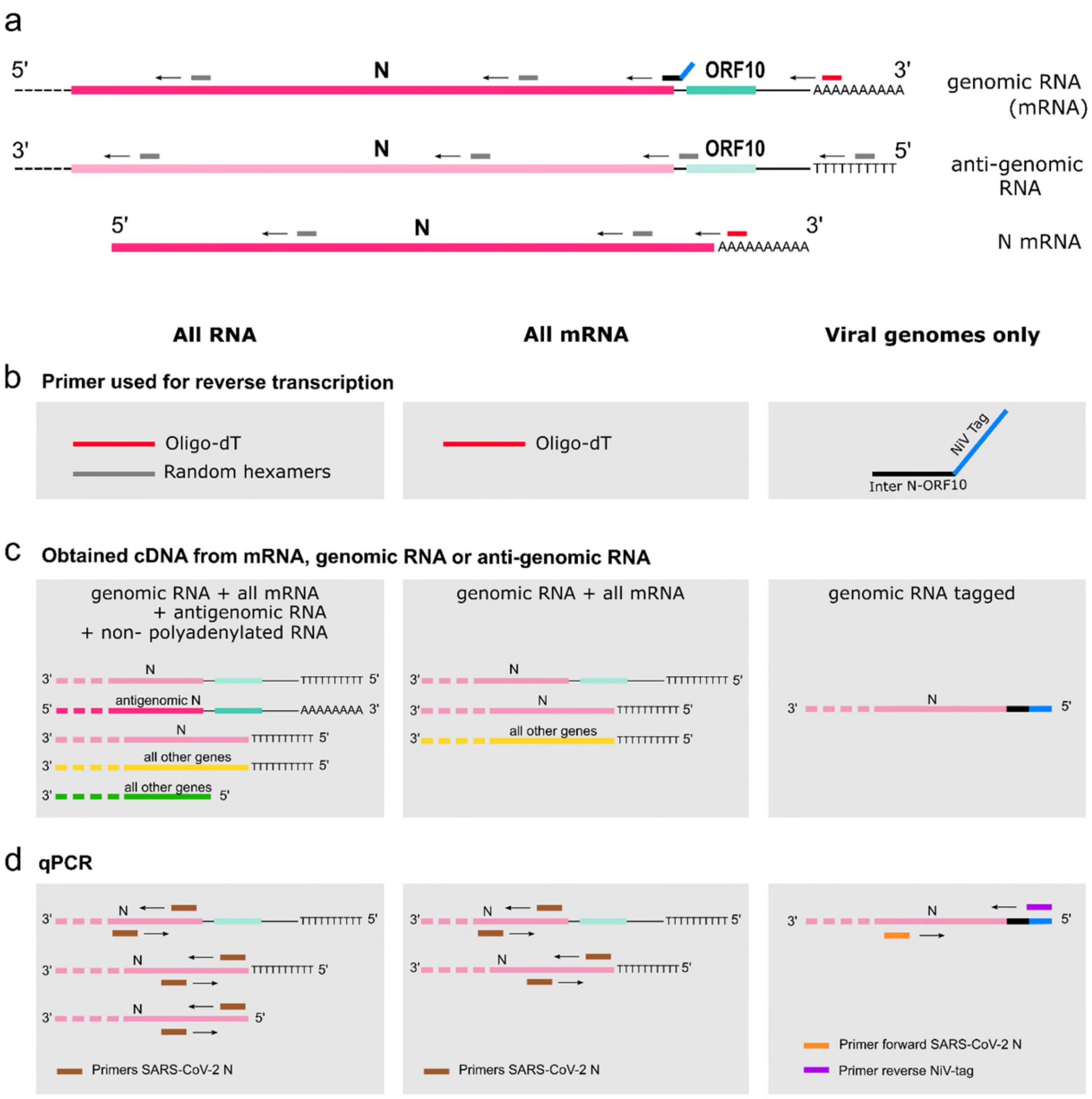

Supplementary figure 6. Schematic of the products obtained by RT-qPCR depending on the reverse transcription method.

a, Zoom on the extremity of SARS-CoV-2 containing the N genomic and anti-genomic RNA and SARS-CoV-2 N mRNA. **b,** primer used to reverse transcribe respectively all RNA, all mRNA or only the genomic RNA. **c,** Overview of the obtained cDNA after the reverse transcription and d, placement of the primer used to amplify SARS-CoV-2 N by qPCR. ORF: open reading frame.

Supplementary Table 1: Titer of viral stocks used

Virus	Titer
BetaCoV/France/IDF0571/2020	$1.3*10^6$ PFU/ml
2019-nCoV/USA_WA1/2020	$1*10^6$ PFU/ml
icSARS-CoV-2-mNG	$1*10^6$ PFU/ml
MeV IC323-EGFP-F L454W	$5*10^5$ PFU/ml
rNiV-EGFP	$4*10^7$ PFU/ml

Supplementary Table 2: Oligonucleotides used for RT-qPCR

Gene	Primer forward	Primer reverse
Hamster_Gapdh	GCATGGCCTTCCGTGTCC	TGTCATCATACTTGGCAGGTTTCT
SARS-CoV-2 genome	GCAGGGCAATCTCACAATCAGG (NiV extension)	CGCATACAAAACATTCCC
SARS-CoV-2 Nucleoprotein	AAACATTCCCACCAACAG	CACTGCTCATGGATTGTT
Hamster_*MX1*	CTTCAAGGAGCACCCACACT	CTTGCCCTCTGGTGACTCTC
Hamster_*TNFa*	TGAGCCATCGTGCCAATG	AGCCCGTCTGCTGGTATCAC
Hamster_*IL6*	GGACAATGACTATGTGTTGTTAGAA	AGGCAAATTTCCCAATTGTATCCAG
Hamster_*CXCL10*	AGACAACAGTAACTCCAGTGACAAG	AGTGTAGCACCTCAGCGTAGC
Hamster_*IL1b*	TGGACCTTCCAGGATGAGGACA	GTTCATCTCGGAGCCTGTAGTG
Hamster_*IL18*	AATGACCCTGGCTGCATTAC	CCAGCATGGGGAGAACTAAA
Hamster_*Gasdermin D*	AACAGGTGACCACGGAAGAC	TGCTGGGTTGGTCATGTAAA
Hamster_*MLKL*	ATCAAGTTTTGCTCGGTGCT	GTGCCTGTATTCCCAGCATT
Hamster_*ISG20*	TGCAGCATTGTGAACTTCAGTG	GCAGGATCTCTAGTCTGGCTTC
Hamster_*CCL5*	ACTGCCTCGTGTTCACATCA	CCCACTTCTTCTTTGGGTTG
Hamster_*Cathepsin* B	GCTGTAATGGTGGCTATC	AGGGAGGTATGGTGTATG
Hamster_*Cathepsin* L	TATGAGGCAAAGGATGGA	TATAGCGACAGCAATAGGT
Hamster_*TMPRSS2*	CTCACTGTGTGGAAGAAC	CTTGGTCTCAGAGTTGTAAT
Hamster_*ACE*2	TCCATTGGTCTTCTGCCATCCG	AGACCATCCACCTCCACTTCTC
Hamster_*Neuropilin-1*	ATAAATGTGGAGGGACTATA	CTGGAGCTTGGATCAGCCAT

Supplementary Table 3: Antibodies used for Immunofluorescent staining

Antibody	Dilution used	Reference
Mouse monoclonal anti-NeuN (clone A-60); Merk	1/200	Cat# MAB377
Mouse monoclonal anti-AQPC (clone D-7); Santa Cruz	1/200	Cat# sc-514022
Mouse monoclonal anti-Myelin Basic Protein [clone MBP101]; Abcam	1/400	Cat# ab62631

Goat polyclonal anti-iba1; Abcam	1/200	Cat# ab48004
Rabbit recombinant anti- SARS-CoV-2 / 2019-nCoV Spike/S2; Sino biological	1/200	Cat# 40590-T62
Goat polyclonal anti-GFAP; Abcam	1/200	Cat# Ab53554
Mouse monoclonal anti-Tubulin, Acetylated (clone 6-11B-1); Sigma-Aldrich	1/1000	Cat# T6793
Mouse monoclonal anti-SARS-CoV-1/2 S Protein (clone 2B3E5); Sigma-Aldrich	1/200	Cat# ZMS1076
Goat polyclonal anti-Olig2; R&D system	1/200	Cat#AF2418
Rabbit polyclonal Anti-Prosurfactant Protein C (proSP-C) ; Millipore	1/100	Cat#AB3786
Alexa FluorTM 488 Goat anti-mouse IgG (H+L); ThermoFisher Scientific	1/500	Cat# A11001
Alexa FluorTM 488 Donkey anti-rabbit IgG (H+L); ThermoFisher Scientific	1/500	Cat# A21206
Alexa FluorTM 555 Donkey anti-mouse IgG (H+L); ThermoFisher Scientific	1/500	Cat# A31570
Alexa FluorTM 555 Donkey anti-rabbit IgG (H+L); ThermoFisher Scientific	1/500	Cat# A31572
Alexa FluorTM 647 Donkey anti-goat IgG (H+L); ThermoFisher Scientific	1/500	Cat# A21447

Supplementary Table 4: Antibodies used for fluorescence-activated single cell sorting

Cell population	Antibody	Fluorochrome	Reference	dilution
Neurons	Monoclonal Anti-GABA A Receptor	PE/Atto 594	Merk / sigma ; SAB5202234-100UG, clone S413-67	1/100
Astrocytes	Anti GLAST (ACSA-1), anti-human/mouse/rat	PE	Miltenyi ; 130-118-344	1/10
Oligodendrocytes	Clone mGalC Anti-Galactocerebroside	Alexa fluor 647	Merk / sigma ; MAB342-AF647	1/100
Microglia	CD68 Rat-Anti-Mouse	BV421	BD ; 566388	1/50
Dead cells	Fixable Viability Dye	eFluor™ 780	Invitrogen™ eBioscience™; 65-0865-14	1/500
